# Withaferin A Induces ROS-Mediated Paraptosis in Human Breast Cancer Cell-Lines MCF-7 and MDA-MB-231

**DOI:** 10.1371/journal.pone.0168488

**Published:** 2016-12-29

**Authors:** Kamalini Ghosh, Soumasree De, Sayantani Das, Srimoyee Mukherjee, Sumita Sengupta Bandyopadhyay

**Affiliations:** Department of Biophysics, Molecular Biology and Bioinformatics, University of Calcutta, India; University of South Alabama Mitchell Cancer Institute, UNITED STATES

## Abstract

Advancement in cancer therapy requires a better understanding of the detailed mechanisms that induce death in cancer cells. Besides apoptosis, themode of other types of cell death has been increasingly recognized in response to therapy. Paraptosis is a non-apoptotic alternative form of programmed cell death, morphologically) distinct from apoptosis and autophagy. In the present study, Withaferin-A (WA) induced hyperpolarization of mitochondrial membrane potential and formation of many cytoplasmic vesicles. This was due to progressive swelling and fusion of mitochondria and dilation of endoplasmic reticulum (ER), forming large vacuolar structures that eventually filled the cytoplasm in human breast cancer cell-lines MCF-7 and MDA-MB-231. The level of indigenous paraptosis inhibitor, Alix/AIP-1 (Actin Interacting Protein-1) was down-regulated by WA treatment. Additionally, prevention of WA-induced cell death and vacuolation on co-treatment with protein-synthesis inhibitor indicated requirement of *de-novo* protein synthesis. Co-treatment with apoptosis inhibitor resulted in significant augmentation of WA-induced death in MCF-7 cells, while partial inhibition in MDA-MB-231 cells; implyingthat apoptosis was not solely responsible for the process.WA-mediated cytoplasmic vacuolationcould not be prevented by autophagy inhibitor wortmanninas well, claiming this process to be a non-autophagic one. Early induction of ROS (Reactive Oxygen Species)by WA in both the cell-lines was observed. ROS inhibitorabrogated the effect of WA on: cell-death, expression of proliferation-associated factor andER-stress related proteins,splicing of XBP-1 (X Box Binding Protein-1) mRNA and formation of paraptotic vacuoles.All these results conclusively indicate thatWA induces deathin bothMCF-7 and MDA-MB-231 cell lines byROS-mediated paraptosis.

## Introduction

Programmed Cell Death (PCD) has been classified into different types based on the biochemical and morphological characteristics of the cells under different pathological and physiological conditions. Type I PCD or apoptosis has been associated with nuclear cell death, which can operate in a caspase-dependent manner [1]. Apoptosis was considered the only way of cancer cell death in the past, but the role of other cellular death mechanisms are being increasingly recognized in response to tumor therapy [2]. Type II PCD or autophagic cell death is mediated by sequestration of cytoplasmic organelles in double or multi-membrane autophagic vesicles and subsequent lysosomal degradation [3]. Type III PCD, characterized by cytoplasmic cell death with non-lysosomal vesiculate [4], also known as paraptosis is a non-apoptotic alternative form of programmed cell death that is ‘related to apoptosis’ but lacks the features that are characteristic of apoptosis (*e*.*g*., nuclear fragmentation, chromatin condensation and the formation of apoptotic bodies) and is insensitive to a broad range of caspase inhibitors [5,6]. The molecular mechanisms of paraptosis induction are not yet well defined. Cross-talk between cell death signaling pathways may allow cells to switch between different mechanisms of PCD. They can activate in parallel and interact [7].

Withaferin A (WA), a naturally occurring steroidal lactone which is derived from the root of *Withaniasomnifera Dunal* (WS) or Ashwagandha, one of the most ancient herbs used in traditional Indian ayurvedic medicine for centuries. Several studies have already demonstrated it as a potent anti-angiogenic agent in a variety of human cancer cells by targeting numerous proteins and modulating their activitiesby directly interacting with them [8]. It was already demonstrated that WA can induce apoptosis as well as autophagy in MCF-7 and MDA-MB-231 cells [9]. However, under similar conditions, cell death in the current study has shown features that are clearly distinct from typical apoptosis or autophagy. Therefore, further experimental evaluations with these cell lines were performed to better characterize programmed cell death under WA treatment.

Paraptosis is characterized by swelling of mitochondria and endoplasmic reticulum (ER), followed by creation of larger vacuoles due to their fusion. Some other features of paraptosis includeabsence of DNA fragmentation, chromatin condensation and PARP cleavage [10]. The appearance of swollen cells suggests ionic deregulation followed by water retention and ultimately disruption of intracellular ion homeostasis causing osmotic lysis, leading to cellular death [11]. Observations, that paraptosis can be inhibited by cycloheximide (CHX), indicate that the process requires protein synthesis [12], thereby distinguishing it from necrosis [4]. The first natural inhibitor of paraptosis, identified by Sperandio *et al*. [13], is known as AIP1/Alix, which is a protein cloned from a calcium-binding protein (ALG-2) involved in T-cell receptor induced cell death. Paraptosis can be activated by MAPK and JNK signaling pathways [13], the TNF receptor family member TAJ/TROY pathways [14] andthe insulin-like growth factor I receptor pathways [10]. Paraptosis appears to be associated with the development of nervous system and neurodegeneration [15]. In addition, various stimuli, like curcumin [12] and ophiobolin-A [16] have been found to induce paraptosis-like cell death in apoptosis-resistant cancer cells. Compounds inhibiting proteasome activity in cancer cells may causeparaptosis-like cell death by inducing unfolded protein response (UPR) and, consequently, ER homeostasis disorder [17–19].

The potency of WA to induce paraptosis in cancer cells has been an untrodden mechanism. Our present study elucidates WA-induced oxidative stress mediated cell death in concert with certain specific characteristics of paraptosis in human breast cancer cell lines MCF-7 and MDA-MB-231.

## Materials and Methods

### Materials

WA was purchased from Alexis Biochemicals, USA. TritonX-100, FBS, DMEM, trypsin-EDTA solution, amphotericin B, penicillin and streptomycin were obtained from Hi-Media, Mumbai, India. DMSO, Wortmannin, Trypan blue, TMRE, Bradford reagent, Luminol, Cycloheximide, Z-vad-fmk, H_2_DCFDA, NAC, P-formaldehyde, PI and HRP-conjugated anti-GAPDH antibody were from SIGMA-Aldrich, USA. Ascorbic acid was purchased from Merck, (Darmstadt, Germany). Cell lysis buffer was purchased from BD Pharmingen, USA. Annexin V-FITC apoptosis detection kit was from Cayman Chemicals, USA. Developer, fixer and X-ray films were from KODAK, Japan. Agar100 resin was obtained from Agar Scientific Co., UK. Trizol was purchased from Invitrogen (CA, USA). All reagents for RT-PCR were from Fermentas (Burlington, Canada). Other chemicals were of analytical grade and purchased locally. β-actin primary antibody and HRP conjugated anti-mouse, anti-rabbit secondary antibodies were obtained from Santacruz, USA. AIP1/Alix and PCNA antibodies were obtained from BioLegend, USA, GRP-78, CHOP antibodies from Cell-Signaling, USA.

### Methods

#### Cell culture and treatments

Human breast epithelial adeno-cancinoma cell-lines, MCF-7 andMDA-MB-231were obtained from National Centre for Cell Science, Pune, India. All these cells were maintained in DMEM supplemented with10% FBS, Amphotericin-B (1.25 μg/ml), penicillin (100 IU/mL) and streptomycin (100 μg/mL) at 37°C in a humidified atmosphere containing 5% CO_2_.Normal human breast epithelial cell line MCF-10A (a kind gift from Prof. P. K. Das, IICB) was used as a control cell line. MCF-10A was maintained in DMEM supplemented with 10% FBS, Amphotericin-B (1.25 μg/ml), penicillin (100 IU/mL) and streptomycin (100 μg/mL), horse serum (5%), Epidermal Growth Factor or EGF (20 ng/ml), hydrocortisone (0.5 mg/ml), cholera toxin (100 ng/ml), insulin (10 μg/ml). Confluent cells (80%) were trypsinized, re-plated (2x10^6^ cells)and allowed 18h for adhesion. Morphology of the cells wasalways observed under microscope (Olympus-CKX41).For treatment of MCF-10A cells, assay medium was used which contained all the supplements except EGF. Cells were treated with WA (4 μM)for 24h or for desired time. Cells were also treated with WT (1 μM)or z-VAD-fmk (25 μM) orcycloheximide (2 μM) or NAC (5mM) or ascorbic acid (100μM) for 24h or time as specified and with H_2_O_2_ (10 mM) for 15 min in presence or absence of WA. Final DMSO concentrations were always maintained below 0.05% (v/v) where no anti-proliferative effects on cells were observed.

#### Cell viability assay

Following treatmentwith NAC (5mM) or CHX (2μM) in presence and absence of WA (4μM) for 24h, MCF-7, MDA-MB-231and MCF-10A cells were collected in PBS(Phosphate buffered saline).Viable cells were counted by staining with trypan blueandIC_50_ values were determined from three independent experiments [20].

#### Measurement of Apoptosis, ROS and mitochondrial membrane potential (Δψm)

For detection of apoptosis, treated cells (2.5×10^6^) werewashed with 1X PBS, stained doubly with FITC conjugated AnnexinV antibody and PI (Propidium Iodide) according to manufacturer’s instructions(Cayman Chemicals) and then subjected to flow-cytometric analysis [BD Fluorescence Activated Cell Sorter (ARIA II), San Jose, CA] using FACS Diva software.

IntracellularROS generation was measured by changes in fluorescence intensity of H_2_DCFDA (excitation480nm, emission 530 nm) by flow-cytometry using cells treated with H_2_DCFDA (10 μM) in dark for 30 min at 37°C.

The changes inMitochondrial Membrane Potential (MMP) of treated cells with respect to the controls (1x10^6^)were detected by exposing cells to different concentrations of WA or equal amount of DMSO (vehicle) for 24h, or 10 mM H_2_O_2_ for 15 min, followed by incubating themwith TMRE (100 nM) for 30 min at 37°C in dark. After washing with PBS, fluorescence intensities(PE-A, 575 nm) of 10,000 cells wereanalyzed by flow cytometry(BD FACS, ARIA II) using FACS Diva software.

#### Western blot

Total cell extracts were prepared from cells (2×10^6^) treated with WA (4 μM) for 24h using lysis buffer (BD Pharmingen) according to manufacturer’s instruction and subjected to immunoblot as described previously [20]. Briefly, blots were incubated with primary antibodies (1:1000) against Alix, PCNA, GRP-78and CHOP,followed by subsequent washing, and then addition of HRP conjugated secondary antibodies (1:5000). After thorough washing with TBS-T, bands were visualized by using Luminol reagents and exposing to X-ray film. β-actin was used as loading control.

#### Transmission electron microscopy

Following treatment; as specified in each case, cells were harvested, washed with PBS and fixed in ice-cold 2.5% glutaraldehyde in PBS, fixed in 1% osmium tetroxide, dehydrated through graded series of ethanol (30%−100%) and embedded in Agar100 resin. After ultrathin sections using anUltracutultramicrotome (Leica) and staining with uranyl acetate (saturated solution) and lead citrate (0.1%), samples were examined under FEI Tecnai 12BioTwin transmission electron microscope.

#### Reverse-Transcription Polymerase Chain Reaction

MCF-7 cells (3x10^6^) were treated with 4 μM of WA in presence and absence of antioxidant NAC (5mM) for 24h and total RNA was extracted using trizol (as manufacturer’s instruction). 2 μg of total RNA was reversed transcribed to prepare cDNAand PCR was performed with specific primers (XBP1: fwd: 5’-CCTTGTAGTTGAGAACCAGG-3’; rev: 5’-GGGGCTTGGTATATATGTGG-3’ and 18S: fwd: 5’-TCAAGAACGAAAGTCGGAGG-3’; rev: 5’-GGACATCTAAGGGCATCAC-3’) using GenAmp PCR system-9700(Applied Biosystems)as described previously[21]. PCR Products were separated in 8% PAGE and after staining with EtBr (0.5% μg/ml), the bands were visualized by Gel Documentation (Bio-Rad).

#### Statistical Analysis

Data are presented as the mean ± standard error (SE) of at least three independent experiments. Statistical analysis of data was conducted by using MS Excel.

## Results

### Extensive cytoplasmic vacuolation is the key feature of WA mediated cell death

**Fig 1:**
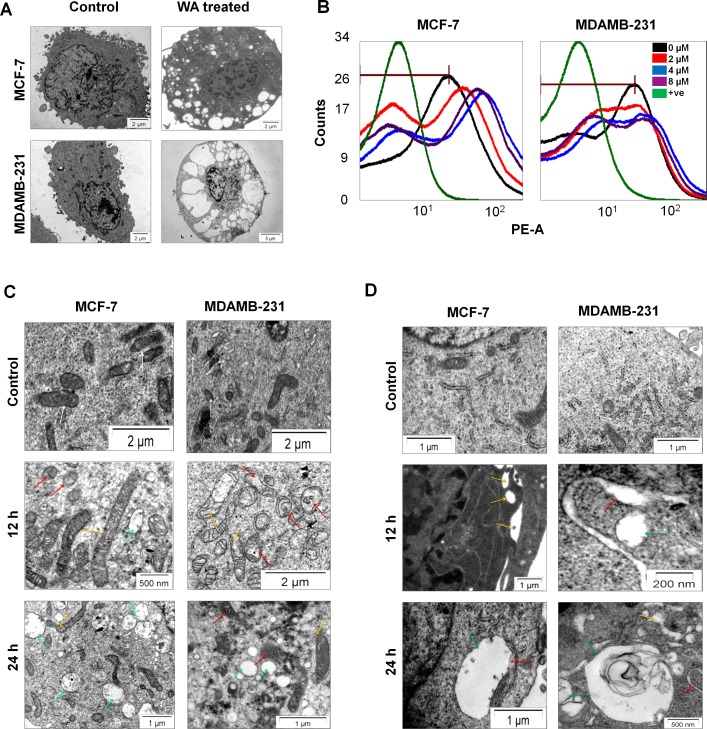
WA induced cytoplasmic vacuolation and altered mitochondrial and ER homeostasis. **(A)** Transmission electron micrographs of MCF-7 and MDA-MB-231 cells, treated with WA (4μM) for 24h showing the ultra-structural morphology of extensive cytoplasmic vacuolation.**(B)** MCF-7 and MDA-MB-231 cells were treated and stained with TMRE as described in method section. The graphsare representative of three individual identical experiments showing mitochondrial hyperpolarization by WA treatment, where “0 μM” represents healthy control cells treated with equal amount of vehicle *i*.*e*. DMSO only for 24h, and “+ve” represents positive control *i*.*e*. cells treated with 10 mM H_2_O_2_for 15 mins for showing mitochondrial depolarization. Both MCF-7 and MDA-MB-231 cells were treated with 0–8 μM of WA for 24h (sample were prepared as described in method section). Electron microscopic images showing the ultrastructural morphology of mitochondria**(C)**; and ER**(D)**; where white, red, yellow and green arrows indicate normal, swelled, enlarged (dilated) and vacuoles respectively.

Ultra-structural analysis of MCF-7 and MDA-MB-231 cells by Transmission Electron Microscope (TEM) demonstrated that treatment with WA for 24h induced extensive cytoplasmicvacuolation in both MCF-7 and MDA-MB-231 cells (Fig 1A).

### WA induced hyper-polarization of MMP (mitochondrial membrane potential)

TMRE is a cell permeable, positively-charged, red-orange fluorescent dye that readily accumulates in active mitochondria due to their relative negative charge and the uptake is directly proportional to the mitochondrial membrane potential (*Δψm*) of cells [22]. Depolarized or inactive mitochondria failed to sequester TMRE that indicate decreased membrane potential. Changes in MMP of MCF-7 and MDA-MB-231 cells treated with WA (0–8 μM) were monitored by flow cytometry using TMRE. The intensities of TMRE fluorescence was continuously augmented byWA treatment for both the cell lines(Fig 1B) as compared to untreated (control), where treatment with H_2_O_2_showeddepolarization of mitochondria. Increased MMP (mitochondrial hyperpolarization) could be indicative of substantial changes in mitochondrial metabolic activity andredox equilibria, which could finally lead to paraptosis.

### WA treatment is associated with morphological changes in mitochondria and Endoplasmic Reticulum

With respect to untreated controls, WA-treated (for 12h) cells displayed both enlarged mitochondria (Fig 1C) and Endoplasmic Reticulum (Fig 1D) as observed under TEM in MCF-7 and MDA-MB-231 cells. Cellular mitochondria were found to be swollen and fused together forming mega-mitochondria (Fig 1C; yellow arrow) with dilated cristae (Fig 1C; red arrow), whereas healthy cells showed mitochondria with fine fibrous distributions(Fig 1C; white arrow). Due to WA treatment, size and number of the ER cisternae first got increased and then dilated (Fig 1D; yellow arrow). Subsequently, fusion among these dilated regions of ER contributed to large dilated empty vacuoles (Fig 1D; green arrow). Interestingly, at 24h post-treatment, most of the ribosomes were found to be no longer appended to the ER membrane (Fig 1D; green arrow). Fusion of mitochondria and ER increased with time until the cells were almost fully occupied by mega-mitochondria and expanded vacuoles (Fig 1A, both MCF-7 and MDA-MB-231 cells). These observations all together resemble the pattern of paraptosis which is characterised by physical enlargement of both mitochondria and ER.

### Suppression of cellular endogenous protein Alix in WA induced cell death

**Fig 2:**
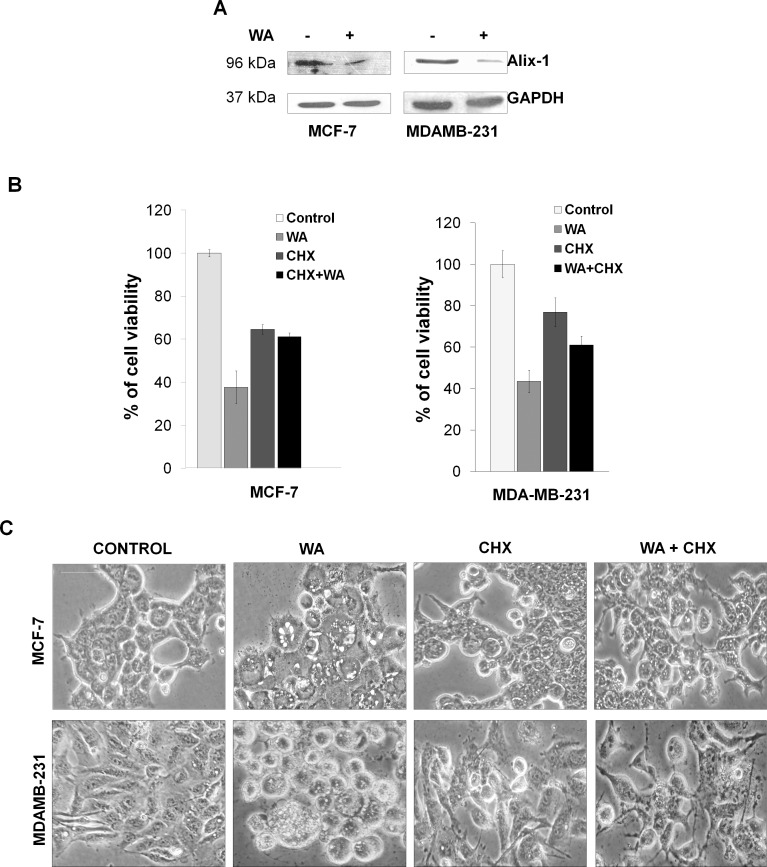
WA induced paraptosis in cells. **(A)** Representative western blot from three separate experiments for Alix-1 protein using cell lysates of MCF-7 and MDA-MB-231 cells treated or untreated with 4μM WA for 24h, where GAPDH used as loading control. **(B)** MCF-7 and MDA-MB-231cells were treated with/without 4 μM of WA for 24h in presence and absence of CHX (2μM) and cell growth inhibition was assessed by Trypan blue exclusion assay. Percentage of viable cells were plotted against drug treatment, where the columns are the mean of three independent determinations; bars, standard error (SEM); (P < 0.05 corresponding to control, n = 3). **(C)** Phase contrast images of MCF-7 and MDA-MB-231 cells, treated with either DMSO or 4 μM of WA for 24h in presence and absence of CHX. Scale bars represent 50 μm and images are representative of at least three independent experiments.

When both MCF-7 and MDA-MB-231 cells were treated with 4μM WA for 24h, significant decrease in the expression of Alix, the known endogenous inhibitor of paraptosis [13] was evident (Fig 2A). Decreased expression of Alix/AIP-1 could be suggestive of induction of paraptosis by WA treatment in both the cell-lines.

### WA-induced cell death was interrupted by translation inhibitor

To check whether new protein synthesis is required for WA mediated cell death, both the breast cancer cell lines were treated for 24h with WA in presence or absence of cyclohexamide (CHX), a translational inhibitor, and cell viability was measured by trypan blue exclusion assay (Fig 2B). Results displayed that co-treatment with cyclohexamide inhibited WA-mediated cell death, indicating requirement of *de-novo* protein synthesis for cellular death that satisfy the necessary requirement for paraptosis.

Microscopicimages of both MCF-7 and MDA-MB-231 cells (Fig 2C) shows extensive cytoplasmic vacuolation with morphological changes upon treatment with 4μM WA for 24h, as compared to respective control cells.These morphological changes and cellular death were abrogated by co-treatment with cyclohexamide,indicating vacuolation, morphological changes and cellular death to be associated with*de-novo* protein synthesis.

### Autophagy inhibitor has no effect on WA-induced cytoplasmic vacuolation

**Fig 3:**
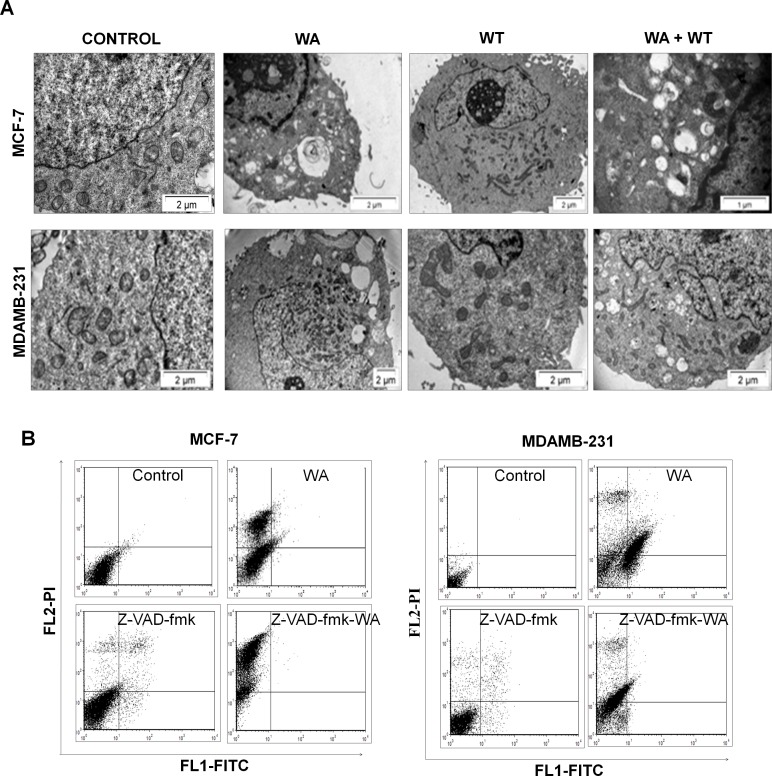
Effect of autophagy and apoptosis inhibitors on WA mediated cell death. **(A)** Transmission electron micrographs of MCF-7 and MDA-MB-231 cells, treated with either DMSO or WA (4 μM) in presence and absence of autophagy inhibitor WT for 24h. **(B)** For apoptosis assay both MCF-7 and MDA-MB-231 cells were either treated with DMSO, WA (4 μM) or pre-treated with zVAD-fmk in presence or absence of WA (4 μM). Cells were harvested after 24h exposure and stained with annexin V-FITC and PI. The samples were analysed using flow cytometer. The percentage of earlyapoptotic, late apoptotic and necrotic cells was located in the lower right (annexin V-FITC positive/PI negative cells), upper right (annexin V-FITC positive/PI positive cells) and upper left (PI positive cells) quadrant respectively.

To distinguish between WA-induced intracellular vacuolation and autophagic cell death, MCF-7 and MDA-MB-231 cells were treated with WA (4 μM)in presence and absence of autophagy inhibitor, WT (1 μM) and observed under TEM. Most strikingly, WT was merely foundto prevent the formation of WA-induced gigantic perinuclear vacuoles (Fig 3A), which established thatautophagy is not entirely responsible for WA-induced cytoplasmic vacuolation for the cell-lines under investigation.

### Sensitivity of human breast cancer cells to WA in presence and absence of pan-caspase inhibitor

To assess the involvement of caspases in WA-induced cell death, MCF-7 and MDA-MB-231 cells were pre-treated with a pan-inhibitor of caspases (z-VAD-fmk) followed by treatment with WA (4 μM) for 24h and the amount of cell death was measured by FACS using annexin/PI double staining method. In the case of MCF-7 cells, 36.8±2.8% cells were found to be necrotic with negligible population of apoptotic cells (Fig 3B), which firmly suggested that apoptosis is not the major mode of WA-mediated cell death in MCF-7 cells. However, in the case of MDA-MB-231 cells, the apoptotic and necrotic populations were 36.6±0.02% and 17.7±3.1% respectively, signifying the fact that MDA-MB-231 cells undergo significant apoptosis on WA treatment. Co-treatment with WA and z-VAD-fmk increased the necrotic population significantly (from 36.8±2.8% to 66.7 ± 1.8%) in MCF-7 cells with no change in the apoptotic population, whereas, in MDA-MB-231 cells, co-treatment decreased the apoptotic population (from 36.6 ± 0.02% to 1.95 ± 0.63%) with concomitant increase in the necrotic population (from 17.7 ± 3.1% to 38.6 ± 1.5%) as compared to cells treated with WA alone. These results indicate that WA induced apoptosis in MDA-MB-231 cells, which was inhibited by addition of caspase inhibitor and the cells underwent caspase-independent cell death. On co-treatment with WA and z-VAD-fmk, increase in cell death (from 39.7 ± 1.9% to 67.6 ± 2.3%) in MCF-7 cells in contrast with partial decrease in total cell death (from 57.5 ± 3.2% to 38.4 ± 2.1%) in MDA-MB-231 cells was clearly evident (refer to left panel of S1 Fig). In conclusion, WA induced death in MDA-MB-231 cells is through both apoptosis and caspase-independent mode; in contrast, death was found to be entirely non-apoptotic and caspase independent in MCF-7 cells.

### WA induced death in breast cancer cells are mediated by ROS pathway

**Fig 4:**
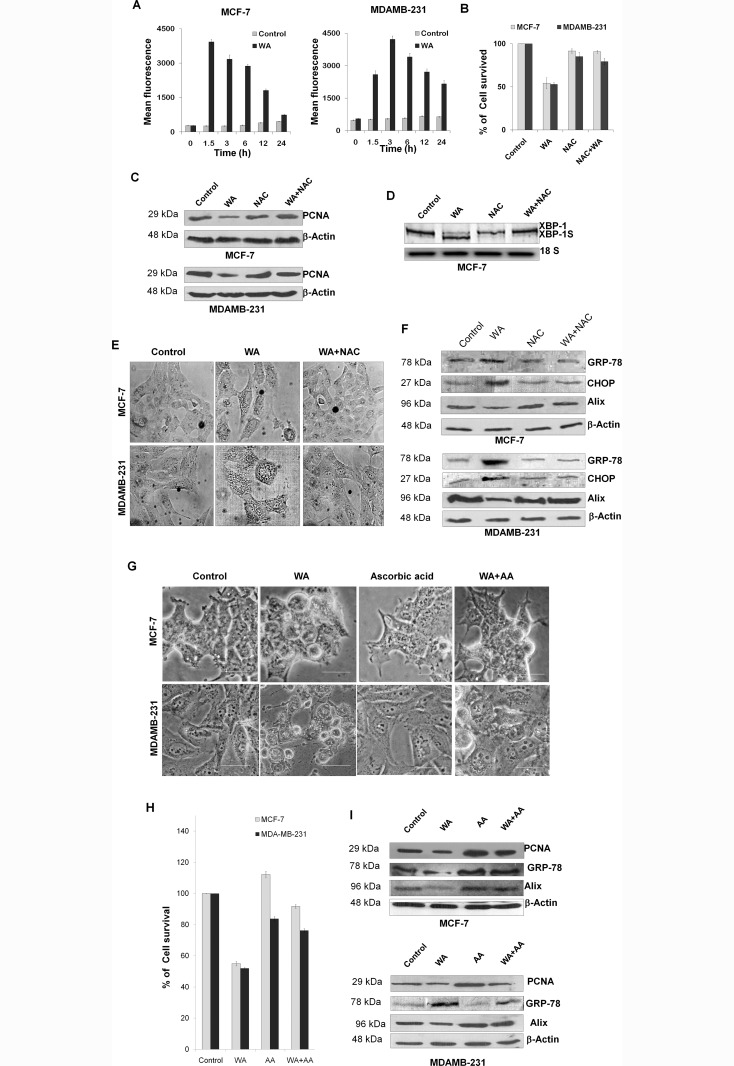
Effect of WA intrinsic ROS generation, ROS mediated ER-stress and paraptosis. **(A)** The inter-cellular ROS generation in WA-treated MCF-7 and MDA-MB-231 cells was measured flow cytometrically by the intensity of H_2_-DCFDA fluorescence. Bar diagram showing mean fluorescence intensity plotted against different time intervals where each point represents as the mean ± SEM of triplicate experiments (P < 0.05 corresponding to control, n = 3). **(B) C**ell growth inhibition ofMCF-7 and MDA-MB-231cells treated with/without WA (4 μM) for 24h in presence and absence of NAC (ROS scavenger) was assessed by Trypan blue exclusion assay. Percentage of viable cells were plotted against drug concentrations, where the columns are the mean of three independent determinations; bars, standard error (SEM); (P < 0.05 corresponding to control, n = 3). **(C)** Representative western blot from three separate experiments for PCNA protein using cell lysates of MCF-7 and MDA-MB-231 cells treated with/without WA (4 μM) for 24h in presence and absence of NAC, where β-actin was used as loading control.**(D)**Total RNA was extracted from MCF-7 cells (treated with/without 4 μM of WA for 24h in presence and absence of NAC) and subjected to RT-PCR where unspliced (XBP1) and spliced (XBP-1s) form of XBP-1 mRNA expression was shown. 18S rRNA was used as loading control.**(E)** Phase contrast images MCF-7 and MDA-MB-231 cells treated (24h) with DMSO or WA (4 μM) in presence or absence of NAC. Scale bars represent 50 micron and images are representative of at least three independent experiments. **(F)** Immune reactive bands of proteins GRP-78, CHOP and Alix extracted fromMCF-7 and MDA-MB-231 cells treated with/without 4 μM of WA for 24h in presence and absence of NAC where GAPDH was used as loading controls.**(G)** Phase contrast images of MCF-7 and MDA-MB-231 cells, treated with either 0 or 4 μM of WA for 24h in presence and absence of ascorbic acid (100 μM). Scale bars represent 50 μm.**(H) C**ell growth inhibition of MCF-7 and MDA-MB-231cells treated with/without WA (4 μM) for 24h in presence and absence of ascorbic acid (another ROS scavenger) was assessed by Trypan blue exclusion assay. Percentage of viable cells were plotted against drug concentrations, where the columns are the mean of three independent determinations; bars, standard error (SEM). **(I)** Western blots from three separate experiments for PCNA, Alix and GRP-78 proteins using cell lysates of MCF-7 and MDA-MB-231 cells treated with/without WA (4 μM) for 24h in presence and absence of ascorbic acid, where β-actin was used as loading control.

WA was found to trigger an early induction of ROS, 1.5h and 3h post treatment for MCF-7 and MDA-MB-231 cells respectively, which gradually decreased over time (Fig 4A). However, no such increase in ROS generation was observed for WA treatment of normal control breast cancer cell line MCF-10A (top left panel of S2 Fig).

Treatment with NAC(5mM), an inhibitor of ROS generation,predominantly inhibited WA-mediated cellular death in MCF-7 and MDA-MB-231 cells(Fig 4B), whereas no significant effect was found in control MCF-10A cells (top right panel of S2 Fig).Down regulation of the level of a proliferation associated protein, PCNA (Fig 4C)was observed for both MCF-7 and MDA-MB-231 *w*.*r*.*t*. WA treated cells. PCNA is a proliferative marker, so this resultsuggests that an early onset of ROS induction was required for WA mediated death or inhibition of proliferation in both the cells.

ROS activates numerous signaling pathways and perform important functions in cells [12]. However, excessive ROS production can induce ER stress-mediated unfolded protein response. Thus, to determine whether WA induced ROS has any role in the induction of UPR in breast cancer cells;cellular RNA was extracted and used for RT-PCR to monitor splicing of XBP-1 mRNA. As shown in Fig 4D, the spliced form of XBP-1 mRNA (*i*.*e*. XBP-1s) was clearly detectable in cells treated with only WA, however this splicing of XBP-1 was found to be abrogated in presence of NAC compared to its respective control.

Further,it was observed under phase contrast microscope that treatment with WA for 12hinduced extensive cytoplasmicvacuolation(Fig 4E), which was abrogated by co-treatment with NACindicating WA-induced early onset of redox imbalance could be responsible for the changes associated with induction of extensive cytoplasmic vacuolation.However, in the case of control MCF-10A cells, no significant vacuolation was observed on WA treatment (Left bottom of S2 Fig).

As shown in Fig 4F, for MCF-7 and MDA-MB-231 cells respectively, the expression levels of ER-stress related proteins like GRP-78 (Bip) and CHOP showed marked elevation by WA treatment which got reduced in presence of NAC, whereas, MCF-10A showed no significant overexpression of GRP-78(right bottom panel of S2 Fig). Furthermore, a significant decrease in the expression of Alix/AIP-1 was found on WA treatment, which was suppressed by co-treatment with NAC, suggestive of the fact that induction of ER-stress by WA is happening through ROS mediated pathway, leading to paraptosis mediated cellular death in both the breast cancer cell-lines.

To rule out the possibility that NAC could be involved in covalent interactions with WA [8], both the cells were treated with another ROS inhibitor, ascorbic acid (100 μM)[23] for 6h, followed by WA treatment and checked cytoplasmic vacuolation, percentage of cell survival and the levels of proliferation associated factorand ER-stress related factors.Phase contrast microscopic images (Fig 4G) showed that treatment with WA for 24 h induced extensive cytoplasmic vacuolation, which was abrogated by co-treatment with ascorbic acid, that corresponds to the earlier observations using NAC. Treatment with ascorbic acid also predominantly inhibited WA-mediated cellular death (Fig 4H), down regulation of PCNA and Alix, alongwith marked elevation ofER-stress related protein GRP-78 (Bip) (Fig 4I). Thus, similar results on treatment with both ROS inhibitors suggest that paraptosis is mediated through early ROS induction by WA in MCF-7 and MDA-MB-231 cells.

## Discussion

Paraptosis is a novel kind of ‘Programmed Cell Death’ (PCD) pathway found to be morphologically and biochemically distinct from apoptosis. This kind of non-apoptotic cell death has been discovered recently and thus its molecular mechanisms have not been fully documented yet. For the last few years, several reportsindicated WA as a potent inducer of apoptosis in diverse human carcinomas [8], but as per our knowledge, no report yet published has presentedthe involvement of WA in paraptotic cell death in any human cancer. Here, we have established for the first time that WA has the capability to induce paraptosis, a non-apoptotic alternative form of PCD in human breast cancer cell-lines.

Apparently, WA elicits extensive cytoplasmic vacuolization in both the cells. Characterization of these WA induced cytoplasmic vacuolization indicated that, it was mostly generated from stress mediated dilation of ER cisternae or swelling and enlargement of mitochondria that bear close resemblance with typical characteristics of paraptosis [10, 13, 14]. Furthermore, cycloheximide (inhibitor of translation) dramatically suppressed the toxicity exerted by WA implying that WA mediated morphological changes and cellular death required*de novo* synthesis of protein [12]. And finally the negative involvement of AIP-1/Alix [13] (endogenous paraptosis inhibitor) protein suggested that WA-induced cell death in case of both MCF-7 and MDA-MB-231 cells is certainly related to paraptosis.

Paraptosis sometimes shows resemblance to necrosis since these two modes of cell death mechanism share some similar kind of morphological features like vacuolization and mitochondrial swelling, along with lack of activation of caspases [10]. It is well known that *in vitro* cell death by caspase dependent apoptosis often results in secondary necrosis[10].In the present study, the role of caspase activation cannot be ruled out totally since z-VAD-fmk was found to increase the PI-positive population significantly in both the cells which could be necrosis.However, when the cells were visualized under TEM, the paraptotic characteristics were evident in both the cells as early as 12h after exposure to WA, long before any dead cells appeared.These results clearly suggested that most of the PI-positive MCF-7 and MDA-MB-231 cells that resulted from WA exposure were committed to paraptosisand not necrosis. In the case of MCF-7 cells no distinctive characteristics of apoptosis, like DNA ladder formation or nuclear condensation and blebbing were observed (refer S3 Fig). However, unlike MCF-7cells,in MDA-MB-231 cells WA induced apoptosis along with paraptosis.Interestinglyit was found that WA-induced vacuolization and cell death in human breast cancer cell-lineswere not affected by autophagyinhibitor,wortmannin, excluding the possibility of autophagy-mediated cell death.

Transmission electron micrographs of WA treated cells revealed severe alteration in mitochondrial morphology (both internal structural features and external shape) implying that mitochondrial swelling could be deemed as a WA induced paraptosis hallmark. MMP depletion generally precedes apoptotic cell death [24]. However, recent studies revealed a key role of mitochondrial permeability transition also in necrosis and mitotic catastrophe [25].Strikingly, WA-induced mitochondrial swelling displayed hyperpolarization of MMP instead of depletion of mitochondrial membrane potential (MMP), suggesting a significant correlation with substantial changes in mitochondrial metabolic homeostasis as well as ionic imbalance as reported earlier [26]. The ER plays an important role in the processing, folding and export of newly synthesized proteins to the secreatory pathway. It has been found that failure of proteasomal degradation system leads to accumulation of misfolded proteins in the ER and cytoplasm. This eventually overwhelms cells and induces ER stress-mediated UPR to protect cells from proteotoxicity [19] but insurmountable disturbance of ER homeostasis may also lead to paraptosis-like cellular death. In a different study, we have found that WA inhibits ubiquitin-mediated proteasomal system and as a consequence, accumulates ubiquitinated proteins over time in case of MCF-7 and MDA-MB-231 cells (unpublished data, not shown here).

Morphological analysis of WA treated cells under TEM revealed that a time-dependent formation of cytoplasmic vacuoles probably derived from ER swelling.At the same time,it also showed dissociationof ribosomes from ER membrane, indicating manifestation of ER-stress mediated UPR that eventually stops new protein synthesis to relieve ER stress [27]. These observations were further paralleled by the levels of XBP-1 spliced mRNA in WA treated cells confirming the concomitant induction of the UPR [28]. Consequently, WA was also found to accumulate ER chaperon proteins in human breast cancer cells (unpublished data; not shown here).

It is worth noting that WA induced paraptosis was associated with a huge induction of oxidative stress in early hours (1.5h-3h) of WA treatment in cells, followed by gradual decrease over time. ROS constitute a crucial group of molecules that mediate numerous signal transduction pathways and perform critical functions in cells [12]. Most cancer cells exhibit increased aerobic glycolysis and oxidative stress compared to those of their normal counterparts [12]. However, an increase in ROS production past a certain level can interfere with ER function, causing the accumulation of large amounts of unfolded or misfolded proteins and leading to the cellular ER stress response [29]. Since WA-mediated formation of cytoplasmic vacuoles, splicing of XBP-1 mRNA, expression of ER-stress related proteinslike GRP78 and CHOP and cellular death were found to be abrogated by co-treatment of cells with the antioxidant NAC as well as ascorbic acid, it could be logical to assume that WA induced paraptotic changes in cells were downstream of the WA mediated ROS generation. Thus, WA induced ER stress could be intimately intertwined with its ability to alter cellular redox balance; however further experimental evidence are required to clarify how WA induced oxidative imbalance influence ER homeostasis which in turn resulted in paraptotic cell death in these human breast cancer cell lines.

Apoptosis resistance is a major obstacle to successful chemotherapy in patients with advanced breast carcinoma and is correlated with the metastatic potential of tumor cells [30]. Conquering this issue is a key goal to re-sensitize tumor cells to cancer therapy by targeting differential mode of programmed cell death pathway(s) [5]. Our results indicated that; in human breast cancer cell-lines MCF-7 and MDA-MB-231, WA induces a novel cytoplasmic vacuolation-mediated cell death (paraptosis), which is distinct from the apoptotic and autophagic cell death pathways. This novel pathway may prevent cancer cells from developing apoptosis or autophagy resistance after the completion of drug treatment. To further understand its mechanisms, we revealed that WA-induced ROS release is an initial signal for the perturbation of ER homeostasis, which subsequently induced cytoplasmic vacuolation mediated cell death. Further investigation of the molecular mechanisms underlying WA-induced paraptosis may lead to the development of a novel therapeutic approach for the more effective management of breast cancercells undergoing death through similar pathways.

## Supporting Information

S1 Fig Effect of caspase inhibitor Z-VAD-fmk on apoptotic cell death in MCF-7 and MDA-MB-231 cells. Apoptotic cell death in WA treated cells by annexin V-FITC/PI double staining method. For apoptosis assay both MCF-7 and MDAMB-231 cells were either treated with DMSO, WA (4 μM) or pre-treated with zVAD-fmk in presence or absence of WA (4 μM). Cells were harvested after 24h exposure and stained with annexin V-FITC and PI. The samples were analysed using flow cytometer. The percentage of total cell death (late apoptotic + necrotic population) was plotted against drug treatments. Each point represented as the mean ± SEM of triplicate experiments (P < 0.05 corresponding to control, n = 3).
(TIF)

S2 Fig Study of ROS generation, cell viability, cellular morphology and expression of ER stress related protein, GRP-78 in control MCF-10A cells. (Top left panel) Graph (representative of three individual identical experiments) showing ROS generation by WA treatment in case of MCF-10A cells. Here “DMSO (control)” represents healthy cells treated with equal amount of vehicle i.e. DMSO for 6h, and “positive control” represents cells treated with 10 mM H_2_O_2_ for 15 mins, otherwise cells were treated with 4 μM of WA for different time periods (as mentioned in the figure). Cells were treated with H_2_DCFDA (10 μM) in dark for 30 min at 37°C and intracellular ROS generation was measured by changes in fluorescence intensity of H_2_DCFDA (excitation 480 nm, emission 530 nm) by flow-cytometry. (Top right panel) **C**ell growth inhibition of MCF-10A cells treated with/without WA (4 μM) for 24h in presence and absence of NAC (ROS scavenger) was assessed by Trypan blue exclusion assay. Percentage of viable cells were plotted against drug concentrations, where the columns are the mean of three independent determinations; bars, standard error (SEM). (Left bottom panel) Phase contrast images of MCF-10A cells, treated with either 0 or 4 μM of WA for 24h in presence and absence of NAC. Scale bars represent 50 μm. (Right bottom panel) Western blot showing expression of GRP-78 of control and WA-treated MCF-10A cells (whole cell extract).
(TIF)

S3 Fig Study of apoptosis in MCF-7 cells by nucleosomal DNA fragmentation and DAPI staining of nuclei on WA treatment. (Left panel) Nucleosomal DNA fragmentation in WA treated MCF-7 cells. Cultured MCF-7 cells were treated with different concentrations of (0–8 μM) WA for 24h. DNA was isolated from each samples and subjected to agarose gel electrophoresis, and visualized by EtBr staining. (Right panel) MCF-7 cells were grown on glass coverslips and were exposed to DMSO or 4 μM of WA for 24h, followed by fixing permeabilized and morphology of nuclei were visualized with an Olympus model CKX41 inverted microscope after staining with DAPI (1 μg/mL) for 30 min in dark.
(TIF)

## Abbreviations

Z-vad-fmk: N-Benzyloxycarbonyl-Val-Ala-Asp(O-Me) fluoromethylketone
Δψm: Mitochondrial membrane potential
WT: Wortmannin
XBP1: X box-binding protein 1
TMRE: Tetramethylrhodamine ethyl ester
H_2_DCFDA: 2', 7'-dichlorodihydrofluorescein diacetate
GAPDH: Glyceraldehyde-3-phosphate dehydrogenase
FITC: Fluorescein isothiocyanate
AIP-1: Actin-interacting protein 1
DMEM: Dulbecco's Modified Eagle's medium
FBS: Fetal bovine serum
HRP: Horseradish peroxidise
EtBr: Ethidium bromide
PAGE: Polyacrylamide gel electrophoresis
PCR: Polymerase chain reaction
PCNA: Proliferating cell nuclear antigen
GRP78: Glucose-regulated protein, 78kDa
CHOP: C/EBP-homologous protein
DMSO: Dimethyl sulfoxide
