# Supplementary figures and images for: Withaferin A Induces ROS-Mediated Paraptosis in Human Breast Cancer Cell-Lines MCF-7 and MDA-MB-231
Source: PLoS One. 2016 Dec 29;11(12):e0168488. doi: 10.1371/journal.pone.0168488 (PMC5199013; doi:10.1371/journal.pone.0168488)

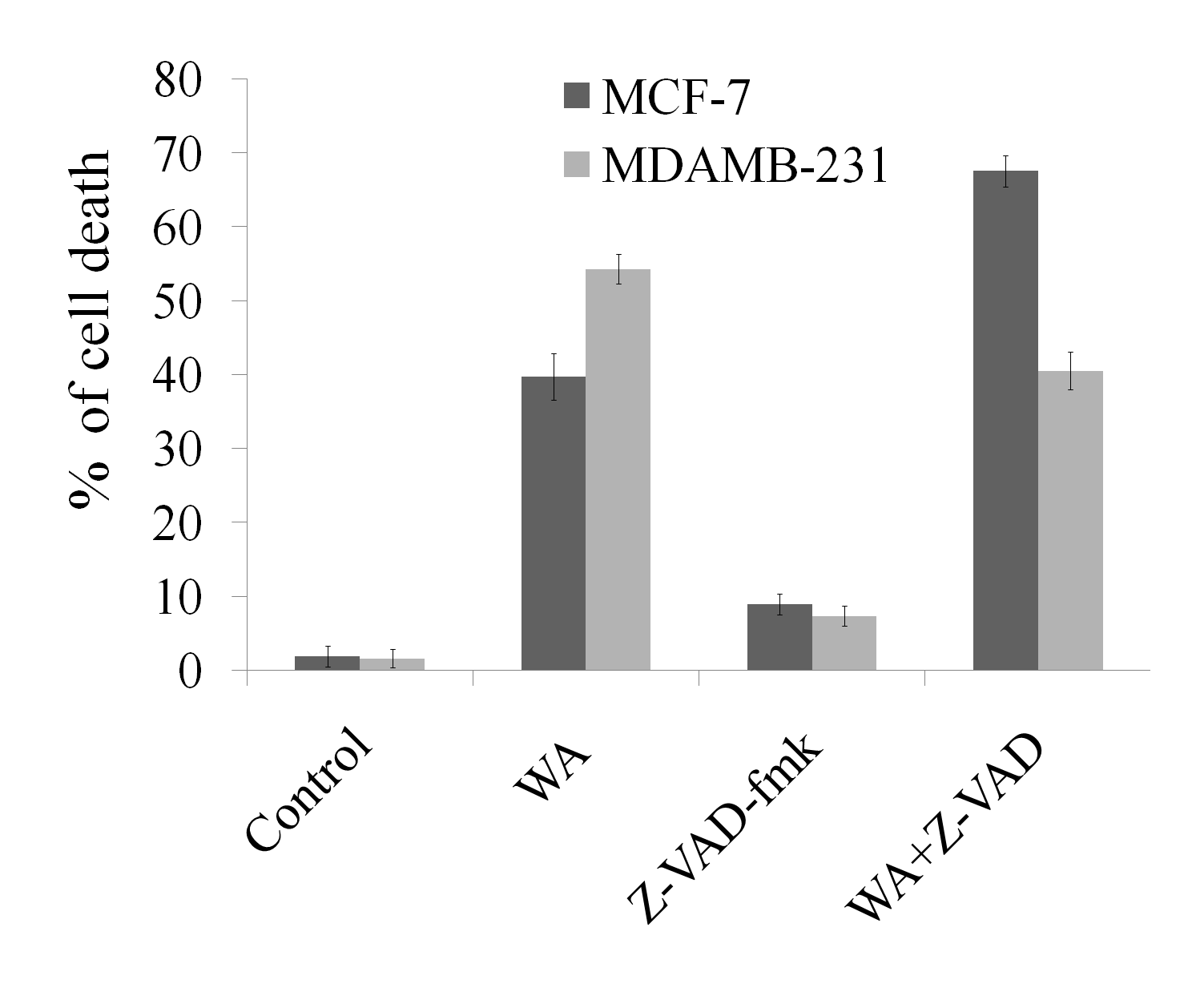

Supplement: S1 Fig — Apoptotic cell death in WA treated cells by annexin V-FITC/PI double staining method. For apoptosis assay both MCF-7 and MDAMB-231 cells were either treated with DMSO, WA (4 μM) or pre-treated with zVAD-fmk in presence or absence of WA (4 μM). Cells were harvested after 24h exposure and stained with annexin V-FITC and PI. The samples were analysed using flow cytometer. The percentage of total cell death (late apoptotic + necrotic population) was plotted against drug treatments. Each point represented as the mean ± SEM of triplicate experiments (P < 0.05 corresponding to control, n = 3). (TIF) [file pone.0168488.s001.tif]

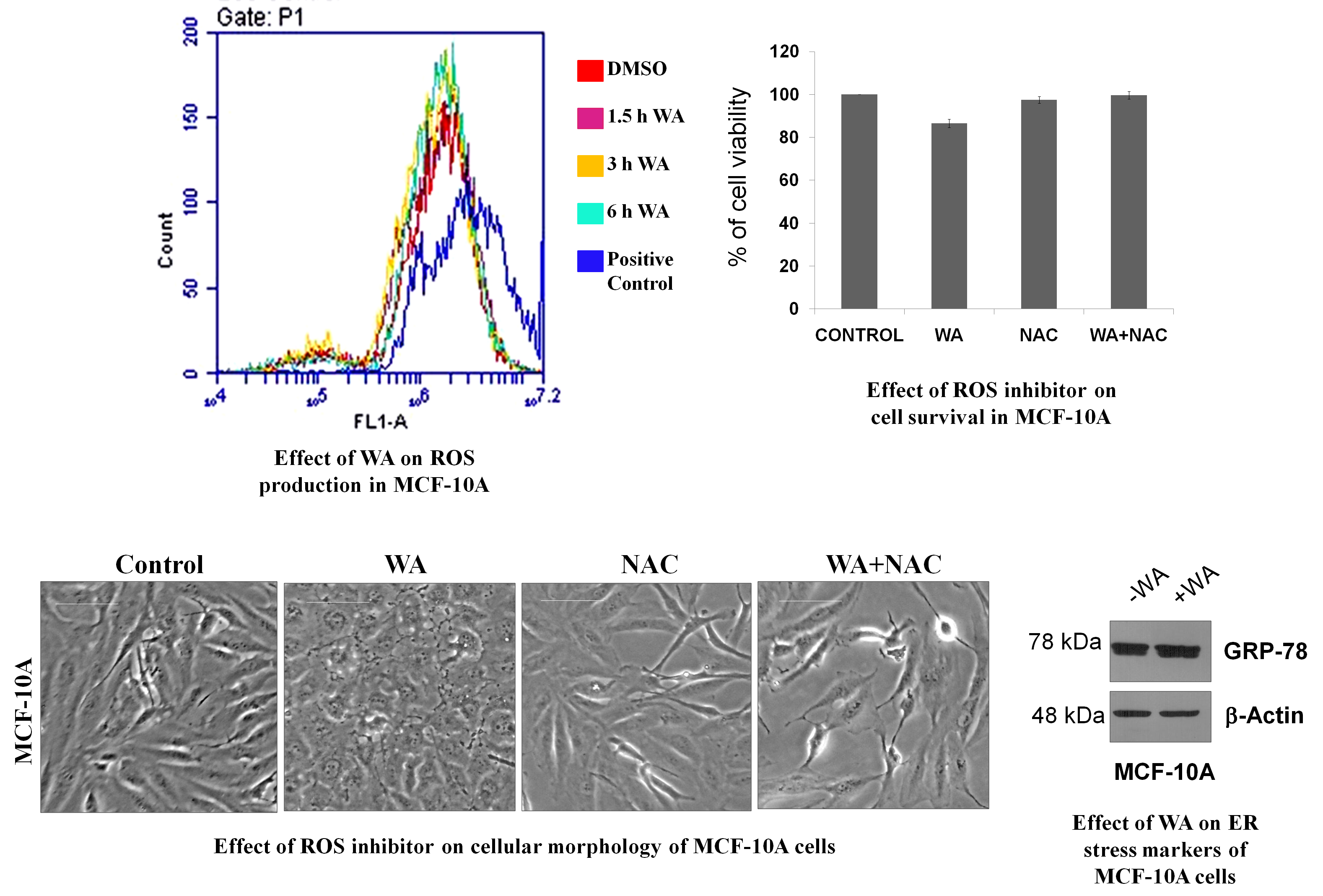

Supplement: S2 Fig — (Top left panel) Graph (representative of three individual identical experiments) showing ROS generation by WA treatment in case of MCF-10A cells. Here “DMSO (control)” represents healthy cells treated with equal amount of vehicle i.e. DMSO for 6h, and “positive control” represents cells treated with 10 mM H2O2 for 15 mins, otherwise cells were treated with 4 μM of WA for different time periods (as mentioned in the figure). Cells were treated with H2DCFDA (10 μM) in dark for 30 min at 37°C and intracellular ROS generation was measured by changes in fluorescence intensity of H2DCFDA (excitation 480 nm, emission 530 nm) by flow-cytometry. (Top right panel) Cell growth inhibition of MCF-10A cells treated with/without WA (4 μM) for 24h in presence and absence of NAC (ROS scavenger) was assessed by Trypan blue exclusion assay. Percentage of viable cells were plotted against drug concentrations, where the columns are the mean of three independent determinations; bars, standard error (SEM). (Left bottom panel) Phase contrast images of MCF-10A cells, treated with either 0 or 4 μM of WA for 24h in presence and absence of NAC. Scale bars represent 50 μm. (Right bottom panel) Western blot showing expression of GRP-78 of control and WA-treated MCF-10A cells (whole cell extract). (TIF) [file pone.0168488.s002.tif]

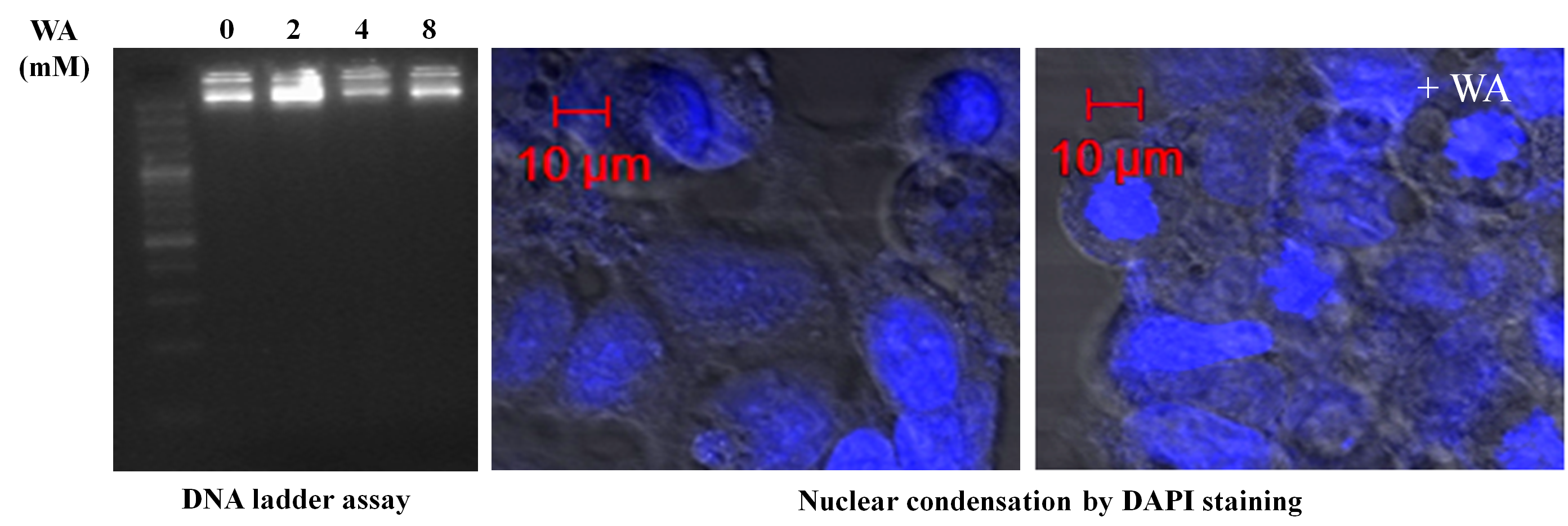

Supplement: S3 Fig — (Left panel) Nucleosomal DNA fragmentation in WA treated MCF-7 cells. Cultured MCF-7 cells were treated with different concentrations of (0–8 μM) WA for 24h. DNA was isolated from each samples and subjected to agarose gel electrophoresis, and visualized by EtBr staining. (Right panel) MCF-7 cells were grown on glass coverslips and were exposed to DMSO or 4 μM of WA for 24h, followed by fixing permeabilized and morphology of nuclei were visualized with an Olympus model CKX41 inverted microscope after staining with DAPI (1 μg/mL) for 30 min in dark. (TIF) [file pone.0168488.s003.tif]
